# Supplementary material for: Morpho-molecular genetic diversity and population structure analysis in garden pea (Pisum sativum L.) genotypes using simple sequence repeat markers
Source: PLoS One. 2022 Sep 16;17(9):e0273499. doi: 10.1371/journal.pone.0273499 (PMC9480992; doi:10.1371/journal.pone.0273499)
Supplement: S1 Table — (DOCX) [file pone.0273499.s001.docx]

**S1 Table. Garden pea evaluated for morpho-molecular study.**

| **No.** | **Name of genotypes** | **Code** | **Source of genotypes** | **Maturity of genotype** | **Pod yield** |
| --- | --- | --- | --- | --- | --- |
| 1. | SP-6 | G1 | CSKHPKV, Palampur | Intermediate | High |
| 2. | SP-3 | G2 | CSKHPKV, Palampur | Intermediate | High |
| 3. | SP-24 | G3 | CSKHPKV, Palampur | Intermediate | Low |
| 4. | SP-22 | G4 | CSKHPKV, Palampur | Intermediate | High |
| 5. | SP-18 | G5 | CSKHPKV, Palampur | Intermediate | Intermediate |
| 6. | SP-12 | G6 | CSKHPKV, Palampur | Intermediate | High |
| 7. | SP-10 | G7 | CSKHPKV, Palampur | Intermediate | High |
| 8. | SN-8-2 | G8 | CSKHPKV, Palampur | Late | High |
| 9. | SN-6 | G9 | CSKHPKV, Palampur | Late | High |
| 10. | SN-5 | G10 | CSKHPKV, Palampur | Intermediate | Intermediate |
| 11. | SN-22 | G11 | CSKHPKV, Palampur | Intermediate | High |
| 12. | SN-2 | G12 | CSKHPKV, Palampur | Intermediate | Low |
| 13. | SN-10 | G13 | CSKHPKV, Palampur | Late | High |
| 14. | DPPM-65 | G14 | CSKHPKV, Palampur | Intermediate | Low |
| 15. | DPPM-74 | G15 | CSKHPKV, Palampur | Late | Low |
| 16. | DPPMFWR-27 | G16 | CSKHPKV, Palampur | Intermediate | Low |
| 17. | DPPMFWR-30 | G17 | CSKHPKV, Palampur | Late | Low |
| 18. | DPPMR-09-1 | G18 | CSKHPKV, Palampur | Late | Low |
| 19. | L-40-1014-1 | G19 | CSKHPKV, Palampur | Intermediate | Low |
| 20. | (PSX19-1)-1 | G20 | CSKHPKV, Palampur | Intermediate | Intermediate |
| 21. | AP-0.3-129 | G21 | CSKHPKV, Palampur | Intermediate | Low |
| 22. | L-0.3-139-1 | G22 | CSKHPKV, Palampur | Late | Low |
| 23. | L-40-1014 | G23 | CSKHPKV, Palampur | Intermediate | Low |
| 24. | L-50-1113-1 | G24 | CSKHPKV, Palampur | Late | Low |
| 25. | Pusa Prabal | G25 | IARI, New Delhi | Intermediate | Low |
| 26. | 2017/PMVAR/1 | G26 | IIVR, Varanasi Under AICRP | Late | Low |
| 27. | 2017/PMVAR/2 | G27 | IIVR, Varanasi Under AICRP | Late | Low |
| 28. | 2017/PMVAR/3 | G28 | IIVR, Varanasi Under AICRP | Late | Intermediate |
| 29. | 2017/PMVAR/4 | G29 | IIVR, Varanasi Under AICRP | Intermediate | Intermediate |
| 30. | 2017/PMVAR/5 | G30 | IIVR, Varanasi Under AICRP | Early | Low |
| 31. | 2017/PMVAR/6 | G31 | IIVR, Varanasi Under AICRP | Early | High |
| 32. | 2017/PMVAR/7 | G32 | IIVR, Varanasi Under AICRP | Intermediate | Low |
| 33. | 2018/PMVAR/1 | G33 | IIVR, Varanasi Under AICRP | Intermediate | High |
| 34. | 2018/PMVAR/2 | G34 | IIVR, Varanasi Under AICRP | Late | Low |
| 35. | 2018/PMVAR/3 | G35 | IIVR, Varanasi Under AICRP | Late | Low |
| 36. | 2018/PMVAR/4 | G36 | IIVR, Varanasi Under AICRP | Intermediate | Low |
| 37. | 2018/PMVAR/5 | G37 | IIVR, Varanasi Under AICRP | Early | Intermediate |
| 38. | 2018/PMVAR/6 | G38 | IIVR, Varanasi Under AICRP | Late | Intermediate |
| 39. | 2018/PMVAR/7 | G39 | IIVR, Varanasi Under AICRP | Intermediate |  |
| 40. | 2018/PMVAR/8 | G40 | IIVR, Varanasi Under AICRP | Intermediate | Low |
| 41. | 2019/PMVAR/1 | G41 | IIVR, Varanasi Under AICRP | Intermediate | High |
| 42. | 2019/PMVAR/2 | G42 | IIVR, Varanasi Under AICRP | Intermediate | Low |
| 43. | 2019/PMVAR/3 | G43 | IIVR, Varanasi Under AICRP | Late | Low |
| 44. | 2019/PMVAR/4 | G44 | IIVR, Varanasi Under AICRP | Early | Low |
| 45. | 2019/PMVAR/5 | G45 | IIVR, Varanasi Under AICRP | Late | Intermediate |
| 46. | 2019/PMVAR/6 | G46 | IIVR, Varanasi Under AICRP | Late | Intermediate |
| 47. | 2019/PMVAR/7 | G47 | IIVR, Varanasi Under AICRP | Intermediate | Intermediate |
| 48. | 2019/PMVAR/8 | G48 | IIVR, Varanasi Under AICRP | Early | Low |
| 49. | Pusa Shree | G49 | IARI, New Delhi | Early | Low |
| 50. | Matar Ageta -6 | G50 | PAU, Ludhiana | Early | Low |
| 51. | Palam Triloki | G51 | CSKHPKV, Palampur | Early | Low |
| 52. | Lincoln | G52 | ICAR-RS, Katrain | Late | Low |
| 53. | Palam Sumool | G53 | CSKHPKV, Palampur | Intermediate | Low |
| 54. | Palam Priya | G54 | CSKHPKV, Palampur | Late | Low |
| 55. | Azad P-1 | G55 | CSAUA&T, Kanpur | Late | Low |
| 56. | Punjab-89 | G56 | PAU, Ludhiana | Intermediate | Intermediate |
