# Supplementary material for: Morpho-molecular genetic diversity and population structure analysis in garden pea (Pisum sativum L.) genotypes using simple sequence repeat markers
Source: PLoS One. 2022 Sep 16;17(9):e0273499. doi: 10.1371/journal.pone.0273499 (PMC9480992; doi:10.1371/journal.pone.0273499)
Supplement: S2 Table — (DOCX) [file pone.0273499.s002.docx]

**S2 Table. List of Primers used in the study**

| **Primer Name** | **Primer sequence** | |
| --- | --- | --- |
|  | **Forward** | **Reverse** |
| **GR01** | CCCAGATGGTAAAGCTACTG | GCTTCATCTGGTGTAGAAGG |
| **GR02** | GTTGAGAGGGAGCTAGTGAA | CTGTCTGATTCTCTCCCTTG |
| **GR06** | ACCCGTCACTACCACTGCTT | GCTAACCTGCTAGGGTCAAC |
| **GR09** | ACCCTAGGAGGGTTTGACAC | CTACCGACGGTGCTGTGTAT |
| **GR25** | GCACCAGCAGCAACTCCT | ACTCTCGGTGCTCCTCTCAC |
| **GR34** | GTATCCAGCAAGAGGCCAAG | CTCCCTCCCTCCTAGGAAAA |
| **GR36** | CCTCCGCAACCTCATACTCC | GACTCACGGTCCCAACTCTC |
| **GR27** | CAACCTCCTCAAGTCGTTAC | GTCCATTGCTCCCTCTACTC |
| **P1109*** | CTCCATCTCAAGAAATCC | CACATAACTAAAAAACCC |
| **Psat5545** | TCCCATGGAACAAGCTCATCATCC | TGGGTTCAGTGAGGAACAGGT |
| **AA339*** | CCAAGAAAGGCTTATCAACAGG | TGCTTGTGTCAAGTGATCAGTG |
| **AB40*** | AAGTGTGAAAGTTTGCCAGGTC | CGGGTACGGGTTATGTTGTC |
| **AB28** | ACGCACACGCTTAGATAGAAAT | ATCCACCATAAGTTTTGGCATA |
| **AA278** | ATTACACCAACAATCTCCCACT | TGTAGAAGCATTTGGGTAGTTG |
| **AA90** | ACAAGACTTCCAGAAATTTTGCAT | AGGACTGATGACGGAGACAAAG |
| **AB68*** | AGCCCAAGTTTCTTCTGAATCC | AAATTCGCAGAGCGTTTGTTAC |
| **AA369*** | GTAAAGCATAAGGGGATTCTCAT | CAGCTTTTAACTCATCTGACACA |
| **AA19** | TCTTTGCTTCCTCATATGTGTC | CGGGATTAAAGTCTCACATTCT |
| **AA92*** | AGCCCAAGTTTCTTCTGAATCC | AAATTCGCAGAGCGTTTGTTAC |
| **AA107** | CCCATGTGAAATTCTCTTGAAGA | GCATTTCACTTGATGAAATTTCG |
| **AA179** | CGCTCACCAAATGTAGATGATAA | TCATGCATCAATGAAAGTGATAAA |
| **AA374** | TCCACCTTCAAAGTTGATACAGT | GGGCTTTTTAGCTTGTAGACCA |
| **AC8** | ATCTCATGTTCAACTTGCAACCTTTA | TTCAAAACACGCAAGTTTTCTGA |
| **D23** | CACATGAGCGTGTGTATGGTAA | GGGATAAGAAGAGGGAGCAAAT |
| **A9** | CCAGATTCATGAAGGGCATACA | GATGAAATTTCGTTTTCTCTGTCTC |
| **D21** | TTGAAGGAACACACAGCGAC | TGCGCACCAAACTACCATAATC |
| **AD147** | CCAAGAAAGGCTTATCAACAGG | TGCTTGTGTCAAGTGATCAGTG |
| **AB72** | AATTAATGCCAATCCTAAGGTATT | GGTTGCACTATTTTCGTTCTC |
| **AB140** | TTTTCACTCAAAACACTCGGCT | GATGCCATTGCTGAAGGAGATT |
| **AD60** | ACAAGACTTCCAGAAATTTTGCAT | AGGACTGATGACGGAGACAAAG |
| **AD51** | TCTTTGCTTCCTCATATGTGTC | CGGGATTAAAGTCTCACATTCT |
| **AB30** | CCCATGTGAAATTCTCTTGAAGA | GCATTTCACTTGATGAAATTTCG |
| **AC22** | CGCTCACCAAATGTAGATGATAA | TCATGCATCAATGAAAGTGATAAA |
| **AA430942** | TCCACCTTCAAAGTTGATACAGT | GGGCTTTTTAGCTTGTAGACCA |
| **AA475** | ATCTCATGTTCAACTTGCAACCTTTA | TCAAAACACGCAAGTTTTCTGA |
| **AC30** | CACATGAGCGTGTGTATGGTAA | GGGATAAGAAGAGGGAGCAAAT |
| **AD158** | CCAGATTCATGAAGGGCATACA | GATGAAATTTCGTTCTCTCTGTCTC |
| **AA335** | TTGAAGGAACACAATCAGCGAC | TGCGCACCAAACTACCATAATC |
| **A5** | AGCATTTGTGCAGTTACAATTTCG | TGATTCACCATCACCATGTGCTAT |
| **AB369** | GATTCTTGAACATCGTGCAGTG | CATTTGAGCTTTCTGGATGACG |
| **AD174*** | GTGCAGAAGCATTTGTTCAGAT | CCCACATATATTTGGTTGGTCA |
| **AA81** | TTAAGATTCTCCATTTTGCCAG | CAACTCGAACTTTAAGGCATTT |
| **AD141** | TAGAGGGTGCTTCCTTCTCAAC | TGGTGGTAAGCAAGTGGGTAGT |
| **AD59** | AACAATAACATGGCAAAGATT | ACCTTGCGATATAATTGATG |
| **AA238** | GTGCATGAAAAAGGGTTCAAGA | CGATGTTGCCATTGATGAAGAC |
| **AA317** | TCAGCCTTTATCCTCCGAACTA | GAACCCTTGTGCAGAAGCATTA |
| **AA160** | AATTTGAAAGAGGCGGATGTG | ACTTCTCTCCAACATCCAACGA |
| **AA416** | GTCAATATCTCCAATGGTAACG | GCATTTGTGTAGTTGTAATTTCAT |
| **AB45*** | CCAACCATTTGTGAGTTCCCTT | TTCGTCGAACCACGAGAATAGA |
| **AB60** | TTTTCACTCAAAACACTCGGCT | GATGCCATTGCTGAAGGAGATT |

* Polymorphic Primer
