# Supplementary material for: Morpho-molecular genetic diversity and population structure analysis in garden pea (Pisum sativum L.) genotypes using simple sequence repeat markers
Source: PLoS One. 2022 Sep 16;17(9):e0273499. doi: 10.1371/journal.pone.0273499 (PMC9480992; doi:10.1371/journal.pone.0273499)
Supplement: S4 Table — (DOCX) [file pone.0273499.s004.docx]

**S 4 Table. Evaluation of garden pea genotypes against powdery mildew disease resistance.**

| **Genotypes** | **Score** | **Characteristics** |
| --- | --- | --- |
| 2017/PMVAR/1, AP-0.3-129 and L-0.3-139-1 | 0 | HR |
| DPPMR-09-1, L-40-1014-1, L-40-1014, L-50-1113-1, Pusa Prabal and Palam Sumool | 1 | R |
| 2017/PMVAR/6, 2018/PMVAR/1, SP-6, SP-3, SP-24, SP-22, SP-18, SP-12, SP-10, SN-8-2, SN-6, SN-5, SN-22, SN-10, DPPMFWR-30, (PSX19-1)-1, Pusa Shree, SN-2 and Pb-89 | 2 | MR |
| DPPM-65, L-50-113-1, 2017/PMVAR/2, 2017/PMVAR/3, 2017/PMVAR/4, 2017/PMVAR/5, 2017/PMVAR/7, 2018/PMVAR/2, 2018/PMVAR/3, 2018/PMVAR/4, 2018/PMVAR/5, 2018/PMVAR/6, 2018/PMVAR/7, 2018/PMVAR/8, 2019/PMVAR/1, 2019/PMVAR/2, 2019/PMVAR/3, 2019/PMVAR/4, 2019/PMVAR/5, 2019/PMVAR/6, 2019/PMVAR/7, 2019/PMVAR/8, Palam Triloki and Palam Priya | 3 | MS |
| Matar Ageta, DPPM-74, DPPMFWR-27, Azad P-1 and Lincoln | 4 | S |

Where, HR-Highly resistant; R-Resistant; MR-Moderately resistant; MS-Moderately Susceptible; S-Susceptible
