# Supplementary material for: Morpho-molecular genetic diversity and population structure analysis in garden pea (Pisum sativum L.) genotypes using simple sequence repeat markers
Source: PLoS One. 2022 Sep 16;17(9):e0273499. doi: 10.1371/journal.pone.0273499 (PMC9480992; doi:10.1371/journal.pone.0273499)
Supplement: S1 Data — (DOCX) [file pone.0273499.s005.docx]

**Data used to draw Fig 1 & Fig 2 (A)**

Morphological characterization of 56 garden pea genotypes

| VARIETIES | Stem: Anthocyanin colouration (Absent/Present) | Foliage: Colour (light green, green, dark green) | Leaf: Leaflets afila type (Absent/Present) | Leaf: Axil colour (Green/Purple) | Stipule: Type (Normal/ Vestigial) | Flower: Opening days (Extra early <40, Early 40-50, Medium 51-70, Late >70) | Flower: Standard petal colour (White, Blue, Pink, Red, purple) | Pod: Number/Axil (Single, Double, Multiple) | Pod: Curvature (Absent, Weak, Medium, Strong) | Pod: Shape of distal part (Pointed/ Blunt) | Pod: Intensity of green colour (Light green, Green, Dark green) | Plant: Height (Short <60, Medium 60-80, Long >80) |
| --- | --- | --- | --- | --- | --- | --- | --- | --- | --- | --- | --- | --- |
| SP-6 | Absent(1) | Green (143B)(5) | Absent(1) | Green(1) | Normal(1) | Late(4) | White(1) | Double(2) | Medium(5) | Pointed(1) | Green (143B)(5) | Medium(5) |
| SP-3 | Absent(1) | Green(143C)(5) | Absent(1) | Green(1) | Normal(1) | Late(4) | White(1) | Double(2) | Weak(3) | Pointed(1) | Green(143C)(5) | Medium(5) |
| SP-24 | Absent(1) | Light green(142B)(3) | Absent(1) | Green(1) | Normal(1) | Late(4) | White(1) | Double(2) | Medium(5) | Pointed(1) | Light green(142B)(3) | Medium(5) |
| SP-22 | Absent(1) | Green(143B)(5) | Absent(1) | Green(1) | Normal(1) | Late(4) | White(1) | Double(2) | Absent(1) | Pointed(1) | Green(143B)(5) | Medium(5) |
| SP-18 | Absent(1) | Green(143B)(5) | Absent(1) | Green(1) | Normal(1) | Late(4) | White(1) | Double(2) | Strong(7) | Pointed(1) | Green(143B)(5) | Medium(5) |
| SP-12 | Absent(1) | Green(143B)(5) | Absent(1) | Green(1) | Normal(1) | Late(4) | White(1) | Double(2) | Medium(5) | Pointed(1) | Green(143B)(5) | Medium(5) |
| SP-10 | Absent(1) | Green(143B)(5) | Absent(1) | Green(1) | Normal(1) | Late(4) | White(1) | Double(2) | Weak(3) | Pointed(1) | Green(143B)(5) | Medium(5) |
| SN-8-2 | Absent(1) | Green(143B)(5) | Absent(1) | Green(1) | Normal(1) | Late(4) | White(1) | Double(2) | Medium(5) | Pointed(1) | Green(143B)(5) | Medium(5) |
| SN-6 | Absent(1) | Green(143B)(5) | Absent(1) | Green(1) | Normal(1) | Late(4) | White(1) | Double(2) | Weak(3) | Pointed(1) | Green(143B)(5) | Medium(5) |
| SN-5 | Absent(1) | Green(143B)(5) | Absent(1) | Green(1) | Normal(1) | Late(4) | White(1) | Double(2) | Weak(3) | Pointed(1) | Green(143B)(5) | Medium(5) |
| SN-22 | Absent(1) | Green(143B)(5) | Absent(1) | Green(1) | Normal(1) | Late(4) | White(1) | Double(2) | Weak(3) | Pointed(1) | Green(143B)(5) | Long(7) |
| SN-2 | Absent(1) | Green(143B)(5) | Absent(1) | Green(1) | Normal(1) | Late(4) | White(1) | Double(2) | Weak(3) | Pointed(1) | Green(143B)(5) | Medium(5) |
| SN-10 | Absent(1) | Green(143B)(5) | Absent(1) | Green(1) | Normal(1) | Late(4) | White(1) | Double(2) | Strong(7) | Pointed(1) | Green(143B)(5) | Medium(5) |
| DPPM-65 | Absent(1) | Dark green(143A)(7) | Absent(1) | Green(1) | Normal(1) | Late(4) | White(1) | Double(2) | Weak(3) | Pointed(1) | Dark green(143A)(7) | Medium(5) |
| DPPM74 | Absent(1) | Green(143B)(5) | Absent(1) | Green(1) | Normal(1) | Late(4) | White(1) | Double(2) | Absent(1) | Blunt(9) | Green(143B)(5) | Long(7) |
| DPPMFWR-27 | Absent(1) | Green(143B)(5) | Absent(1) | Green(1) | Normal(1) | Late(4) | White(1) | Double(2) | Weak(3) | Pointed(1) | Green(143B)(5) | Long(7) |
| DPPMFWR-30 | Absent(1) | Green(143B)(5) | Absent(1) | Green(1) | Normal(1) | Late(4) | White(1) | Multiple(3) | Medium(5) | Pointed(1) | Green(143B)(5) | Medium(5) |
| DPPMR-09-1 | Absent(1) | Green(143B)(5) | Absent(1) | Green(1) | Normal(1) | Late(4) | White(1) | Double(2) | Absent(1) | Blunt(9) | Green(143B)(5) | Medium(5) |
| L-40-1014-1 | Absent(1) | Light green(142B)(3) | Absent(1) | Green(1) | Normal(1) | Late(4) | White(1) | Double(2) | Medium(5) | Pointed(1) | Light green(142B)(3) | Medium(5) |
| (PSX19-1)-1 | Absent(1) | Green(143B)(5) | Absent(1) | Green(1) | Normal(1) | Late(4) | White(1) | Double(2) | Medium(5) | Pointed(1) | Green(143B)(5) | Medium(5) |
| AP-0.3-129 | Absent(1) | Green(143B)(5) | Absent(1) | Green(1) | Normal(1) | Late(4) | White(1) | Double(2) | Absent(1) | Blunt(9) | Green(143B)(5) | Medium(5) |
| L-0.3-139-1 | Absent(1) | Dark green(143A)(7) | Absent(1) | Green(1) | Normal(1) | Late(4) | White(1) | Double(2) | Weak(3) | Pointed(1) | Dark green(143A)(7) | Medium(5) |
| L-40-1014 | Absent(1) | Green(143B)(5) | Absent(1) | Green(1) | Normal(1) | Late(4) | White(1) | Double(2) | Medium(5) | Pointed(1) | Green(143B)(5) | Long(7) |
| L-50-1113-1 | Absent(1) | Green(143B)(5) | Absent(1) | Green(1) | Normal(1) | Late(4) | White(1) | Double(2) | Medium(5) | Pointed(1) | Green(143B)(5) | Medium(5) |
| Pusa Prabal | Absent(1) | Green(143B)(5) | Absent(1) | Green(1) | Normal(1) | Late(4) | White(1) | Double(2) | Absent(1) | Blunt(9) | Green(143B)(5) | Medium(5) |
| 2017/PMVAR/1 | Absent(1) | Light green(142B)(3) | Absent(1) | Green(1) | Normal(1) | Late(4) | White(1) | Double(2) | Medium(5) | Pointed(1) | Light green(142B)(3) | Medium(5) |
| 2017/PMVAR/2 | Absent(1) | Green(143B)(5) | Absent(1) | Green(1) | Normal(1) | Late(4) | White(1) | Double(2) | Medium(5) | Pointed(1) | Green(143B)(5) | Long(7) |
| 2017/PMVAR/3 | Absent(1) | Green(143B)(5) | Absent(1) | Green(1) | Normal(1) | Late(4) | White(1) | Double(2) | Weak(3) | Pointed(1) | Green(143B)(5) | Long(7) |
| 2017/PMVAR/4 | Absent(1) | Green(143B)(5) | Absent(1) | Green(1) | Normal(1) | Late(4) | White(1) | Double(2) | Weak(3) | Pointed(1) | Green(143B)(5) | Long(7) |
| 2017/PMVAR/5 | Absent(1) | Green(143B)(5) | Absent(1) | Green(1) | Normal(1) | Late(4) | White(1) | Double(2) | Weak(3) | Pointed(1) | Green(143B)(5) | Medium(5) |
| 2017/PMVAR/6 | Absent(1) | Green(143B)(5) | Absent(1) | Green(1) | Normal(1) | Late(4) | White(1) | Double(2) | Absent(1) | Blunt(9) | Green(143B)(5) | Medium(5) |
| 2017/PMVAR/7 | Absent(1) | Green(143B)(5) | Absent(1) | Green(1) | Normal(1) | Late(4) | White(1) | Double(2) | Weak(3) | Pointed(1) | Green(143B)(5) | Long(7) |
| 2018/PMVAR/1 | Absent(1) | Green(143B)(5) | Absent(1) | Green(1) | Normal(1) | Late(4) | White(1) | Double(2) | Medium(5) | Pointed(1) | Green(143B)(5) | Long(7) |
| 2018/PMVAR/2 | Absent(1) | Green(143B)(5) | Absent(1) | Green(1) | Normal(1) | Late(4) | White(1) | Double(2) | Weak(3) | Pointed(1) | Green(143B)(5) | Long(7) |
| 2018/PMVAR/3 | Absent(1) | Light green(142B)(3) | Absent(1) | Green(1) | Normal(1) | Late(4) | White(1) | Double(2) | Weak(3) | Pointed(1) | Light green(142B)(3) | Long(7) |
| 2018/PMVAR/4 | Absent(1) | Green(143B)(5) | Absent(1) | Green(1) | Normal(1) | Late(4) | White(1) | Double(2) | Absent(1) | Blunt(9) | Green(143B)(5) | Long(7) |
| 2018/PMVAR/5 | Absent(1) | Green(143B)(5) | Absent(1) | Green(1) | Normal(1) | Late(4) | White(1) | Double(2) | Weak(3) | Pointed(1) | Green(143B)(5) | Medium(5) |
| 2018/PMVAR/6 | Absent(1) | Light green(142B)(3) | Absent(1) | Green(1) | Normal(1) | Late(4) | White(1) | Double(2) | Medium(5) | Pointed(1) | Light green(142B)(3) | Long(7) |
| 2018/PMVAR/7 | Absent(1) | Light green(142B)(3) | Absent(1) | Green(1) | Normal(1) | Late(4) | White(1) | Double(2) | Weak(3) | Pointed(1) | Light green(142B)(3) | Long(7) |
| 2018/PMVAR/8 | Absent(1) | Green(143B)(5) | Absent(1) | Green(1) | Normal(1) | Late(4) | White(1) | Double(2) | Weak(3) | Pointed(1) | Green(143B)(5) | Long(7) |
| 2019/PMVAR/1 | Absent(1) | Green(143B)(5) | Absent(1) | Green(1) | Normal(1) | Late(4) | White(1) | Double(2) | Weak(3) | Pointed(1) | Green(143B)(5) | Long(7) |
| 2019/PMVAR/2 | Absent(1) | Green(143B)(5) | Absent(1) | Green(1) | Normal(1) | Late(4) | White(1) | Double(2) | Absent(1) | Blunt(9) | Green(143B)(5) | Medium(5) |
| 2019/PMVAR/3 | Absent(1) | Green(143B)(5) | Absent(1) | Green(1) | Normal(1) | Late(4) | White(1) | Double(2) | Weak(3) | Pointed(1) | Green(143B)(5) | Long(7) |
| 2019/PMVAR/4 | Absent(1) | Light green(142B)(3) | Absent(1) | Green(1) | Normal(1) | Late(4) | White(1) | Double(2) | Weak(3) | Pointed(1) | Light green(142B)(3) | Medium(5) |
| 2019/PMVAR/5 | Absent(1) | Green(143B)(5) | Absent(1) | Green(1) | Normal(1) | Late(4) | White(1) | Double(2) | Weak(3) | Pointed(1) | Green(143B)(5) | Long(7) |
| 2019/PMVAR/6 | Absent(1) | Green(143B)(5) | Absent(1) | Green(1) | Normal(1) | Late(4) | White(1) | Double(2) | Absent(1) | Blunt(9) | Green(143B)(5) | Long(7) |
| 2019/PMVAR/7 | Absent(1) | Green(143B)(5) | Absent(1) | Green(1) | Normal(1) | Late(4) | White(1) | Double(2) | Weak(3) | Pointed(1) | Green(143B)(5) | Medium(5) |
| 2019/PMVAR/8 | Absent(1) | Light green(142B)(3) | Present(9) | Green(1) | Normal(1) | Late(4) | White(1) | Double(2) | Weak(3) | Pointed(1) | Light green(142B)(3) | Medium(5) |
| Pusa Shree | Absent(1) | Light green(142A)(3) | Absent(1) | Green(1) | Normal(1) | Medium(3) | White(1) | Double(2) | Absent(1) | Blunt(9) | Light green(142A)(3) | Medium(5) |
| Matar Ageta | Absent(1) | Green(143B)(5) | Absent(1) | Green(1) | Normal(1) | Medium(3) | White(1) | Double(2) | Weak(3) | Pointed(1) | Green(143B)(5) | Medium(5) |
| Palam Triloki | Absent(1) | Green(143D)(5) | Absent(1) | Green(1) | Normal(1) | Medium(3) | White(1) | Double(2) | Strong(7) | Pointed(1) | Green(143D)(5) | Medium(5) |
| Lincoln | Absent(1) | Green(143B)(5) | Absent(1) | Green(1) | Normal(1) | Late(4) | White(1) | Double(2) | Medium(5) | Pointed(1) | Green(143B)(5) | Long(7) |
| Palam Sumool | Absent(1) | Green(143B)(5) | Absent(1) | Green(1) | Normal(1) | Late(4) | White(1) | Double(2) | Weak(3) | Pointed(1) | Green(143B)(5) | Medium(5) |
| Palam Priya | Absent(1) | Green(143C)(5) | Absent(1) | Green(1) | Normal(1) | Late(4) | White(1) | Double(2) | Weak(3) | Pointed(1) | Green(143C)(5) | Medium(5) |
| Azad P-1 | Absent(1) | Green(143B)(5) | Absent(1) | Green(1) | Normal(1) | Late(4) | White(1) | Double(2) | Medium(5) | Pointed(1) | Green(143B)(5) | Medium(5) |
| Pb-89 | Absent(1) | Green(143D)(5) | Absent(1) | Green(1) | Normal(1) | Late(4) | White(1) | Double(2) | Absent(1) | Blunt(9) | Green(143D)(5) | Medium(5) |

**Data used to draw Fig 1:**

Characterization of 56 diverse garden pea genotypes based on DUS as per PPV&FRA

| Trait | Class or scale of descriptor | Distribution by classes of descriptor (%) |
| --- | --- | --- |
| Stem: Anthocyanin colouration | Absent | 56 (100%) |
|  | Present | - |
| Foliage colour | Light green | 9 (16.07%) |
|  | Green | 45 (80.35%) |
|  | Dark green | 2 (3.57%) |
| Leaf: Leaflets afila type | Absent | 55 (98.21%) |
|  | Present | 1 (1.78%) |
| Leaf: Axil colour | Green | 56 (100%) |
|  | Purple | - |
| Stipule type | Normal | 56 (100%) |
|  | Vestigial | - |
| Flower opening days | Extra early <40 days | - |
|  | Early 40-50 days | - |
|  | Medium 51-70 days | 3 (5.35%) |
|  | Late >70 days | 53 (94.64%) |
| Flower: Standard petal colour | White | 56 (100%) |
|  | Blue | - |
|  | Pink | - |
|  | Red | - |
|  | Purple | - |
| Pod number per axil | Single | - |
|  | Double | 55 (98.21%) |
|  | Multiple | 1 (1.78%) |
| Pod curvature | Absent | 11 (19.64%) |
|  | Weak | 27 (48.21%) |
|  | Medium | 15 (26.78%) |
|  | Strong | 3 (5.35%) |
| Pod: Shape of distal part | Pointed | 45 (80.35%) |
|  | Blunt | 11 (19.64%) |
| Pod: Intensity of green colour | Light green | 9 (16.07%) |
|  | Green | 45 (80.35%) |
|  | Dark green | 2 (3.57%) |
| Plant height | Short <60cm | - |
|  | Medium 60-80cm | 36 (64.28%) |
|  | Long >80cm | 20 (35.71%) |

**Data used to draw Fig 3**

**PCA of 19 morphological traits with eigenvalues, variability and cumulative variances (pooled).**

|  | **PC1** | **PC2** | **PC3** | **PC4** | **PC5** | **PC6** | **PC7** |
| --- | --- | --- | --- | --- | --- | --- | --- |
| Days to 50% flowering | -0.065 | 0.462 | -0.073 | -0.117 | -0.140 | 0.064 | -0.035 |
| First Flower Node | -0.124 | 0.425 | -0.042 | -0.042 | 0.084 | 0.084 | 0.005 |
| Days to first picking | -0.043 | 0.494 | 0.030 | -0.063 | -0.062 | 0.054 | -0.095 |
| Number of branches | 0.227 | 0.105 | -0.108 | 0.535 | 0.047 | 0.118 | 0.327 |
| Internodal length (cm) | 0.181 | 0.183 | 0.140 | 0.021 | 0.482 | -0.356 | 0.061 |
| Nodes per plant | 0.234 | 0.261 | -0.079 | 0.429 | 0.079 | 0.134 | 0.244 |
| Plant height(cm) | 0.049 | 0.231 | -0.053 | -0.181 | 0.533 | -0.266 | 0.057 |
| Pod length(cm) | 0.375 | 0.109 | 0.146 | -0.261 | -0.107 | -0.092 | -0.091 |
| Pod width(cm) | 0.112 | -0.028 | 0.561 | 0.089 | 0.104 | 0.192 | -0.008 |
| Seeds per pod | 0.410 | 0.006 | -0.094 | -0.277 | -0.058 | -0.035 | -0.083 |
| Shelling (%) | 0.210 | 0.015 | -0.367 | 0.100 | -0.019 | 0.298 | -0.349 |
| Pods per plant | 0.262 | -0.027 | -0.454 | -0.063 | 0.035 | 0.009 | -0.078 |
| Pod yield per plant(g) | 0.451 | -0.039 | -0.115 | -0.107 | -0.033 | 0.023 | -0.071 |
| Average pod weight(g) | 0.366 | -0.013 | 0.399 | -0.071 | -0.105 | 0.019 | 0.022 |
| Harvest duration (days) | 0.185 | -0.368 | -0.068 | 0.035 | 0.101 | -0.146 | 0.286 |
| Moisture content (%) | -0.136 | -0.173 | -0.184 | -0.106 | 0.471 | 0.255 | 0.089 |
| Total soluble solids (⸰Brix) | 0.082 | 0.042 | 0.113 | 0.441 | -0.106 | -0.155 | -0.284 |
| Ascorbic acid (mg) | 0.079 | 0.007 | 0.174 | -0.196 | 0.184 | 0.710 | 0.132 |
| Sugar content (mg/g) | 0.011 | 0.118 | -0.110 | -0.220 | -0.359 | -0.073 | 0.692 |
| Eigenvalue | 4.066 | 3.452 | 2.065 | 1.546 | 1.370 | 1.230 | 1.052 |
| Variability (%) | 21.398 | 18.171 | 10.869 | 8.136 | 7.210 | 6.475 | 5.535 |
| Cumulative % | 21.398 | 39.569 | 50.439 | 58.575 | 65.785 | 72.260 | 77.795 |
