# Supplementary material for: Morpho-molecular genetic diversity and population structure analysis in garden pea (Pisum sativum L.) genotypes using simple sequence repeat markers
Source: PLoS One. 2022 Sep 16;17(9):e0273499. doi: 10.1371/journal.pone.0273499 (PMC9480992; doi:10.1371/journal.pone.0273499)
Supplement: S1 File — (DOCX) [file pone.0273499.s009.docx]

**Table -1S: Custer compositions in garden pea following multivariate analysis in 2019-20, 2020-21 and pooled over years**

| **Cluster number** | **No. of genotypes** | **Genotype** |
| --- | --- | --- |
| **2019-20** | | |
| I | 14 | SP-18,SN-6,2017/PMVAR/6,2018/PMVAR/1,2019/PMVAR/1,2019/PMVAR/7,SN-5,SP-10,Pb-89,SN-10,2017/PMVAR/5,2018/PMVAR/5,SP-24,DPPM-65 |
| II | 5 | SP-6,SP-3,SP-22,SP-12,SN-8-2 |
| III | 28 | DPPMR-09-1,Lincoln,AP-0.3-129,L-0.3-139-1,DPPM-74,L-50-1113-1,Azad P-1,Pusa Prabal,L-40-1014-1,2019/PMVAR/6,Palam Priya,2017/PMVAR/3,2019/PMVAR/5,2018/PMVAR/2,2019/PMVAR/3,L-40-1014,2019/PMVAR/2,2018/PMVAR/8,2017/PMVAR/7,2017/PMVAR/2,2017/PMVAR/4,2018/PMVAR/7,(PSX19-1)-1,2018/PMVAR/4,DPPMFWR-27,SN-2,SN-22,2018/PMVAR/6 |
| IV | 5 | Matar Ageta,Palam Triloki,Pusa Shree,2019/PMVAR/8,2019/PMVAR/4 |
| V | 1 | 2017/PMVAR/1 |
| VI | 1 | DPPMFWR-30 |
| VII | 1 | 2018/PMVAR/3 |
| VIII | 1 | Palam Sumool |
| **2020-21** | | |
| I | 30 | SP-6,2018/PMVAR/1,SP-3,2019/PMVAR/1,SP-22,SN-10,2017/PMVAR/6,2018/PMVAR/5,SP-10,SP-12,2018/PMVAR/7,SN-8-2,2019/PMVAR/5,2018/PMVAR/6,SN-22,Pb-89,SN-6,(PSX19-1)-1,2017/PMVAR/4,2017/PMVAR/7,SP-24,2018/PMVAR/4,L-50-1113-1,2019/PMVAR/7,2019/PMVAR/2,2018/PMVAR/2,2017/PMVAR/2,2017/PMVAR/5,2019/PMVAR/4,SN-5 |
| II | 18 | 2018/PMVAR/3,2019/PMVAR/3,Azad P-1,DPPM-65,2017/PMVAR/1,Lincoln,DPPMR-09-1,DPPMFWR-30,Pusa Prabal,DPPM-74,DPPMFWR-27,L-40-1014,L-40-1014-1,2018/PMVAR/8,AP-0.3-129,2017/PMVAR/3,L-0.3-139-1,2019/PMVAR/8 |
| III | 6 | Pusa Shree,Matar Ageta,Palam Triloki,Palam Piya,SN-2,SP-18 |
| IV | 1 | 2019/PMVAR/6 |
| V | 1 | Palam Sumool |
| **Pooled** | | |
| I | 30 | 2018/PMVAR/1,2019/PMVAR/1,SP-22,2017/PMVAR/6,SP-3,SP-6,SP-12,SP-10,SN-6,SN-10,Pb-89,2018/PMVAR/5,SN-8-2,2018/PMVAR/7,SP-18,SP-24,2019/PMVAR/7,SN-5,2019/PMVAR/5,SN-22,2017/PMVAR/5,(PSX19-1)-1,2017/PMVAR/4,2019/PMVAR/2,L-50-1113-1,2018/PMVAR/4,2018/PMVAR/6,DPPM-65,2017/PMVAR/7,SN-2 |
| II | 1 | Azad P-1 |
| III | 20 | DPPMR-09-1,Lincoln,DPPM-74,AP-0.3-129,DPPMFWR-30,L-0.3-139-1,2017/PMVAR/1,2018/PMVAR/8,2017/PMVAR/2,Pusa Prabal,L-40-1014-1,2019/PMVAR/3,2019/PMVAR/6,L-40-1014,2017/PMVAR/3,2018/PMVAR/2,2018/PMVAR/3,DPPMFWR-27,2019/PMVAR/8,Palam Priya |
| IV | 3 | Pusa Shree,Matar Ageta,Palam Triloki |
|  |  |  |

**Table -2S-I: Average intra and inter-cluster distances in garden pea during 2019-20**

| Clusters | I | II | III | IV | V | VI | VII | VIII |
| --- | --- | --- | --- | --- | --- | --- | --- | --- |
| I | **5.84** | 7.51 | 8.37 | 8.98 | 10.02 | 11.68 | 11.03 | 9.05 |
|  | **(2.42)** | (2.74) | (2.89) | (3.00) | (3.17) | (3.42) | (3.32) | (3.01) |
| II |  | **5.45** | 11.73 | 12.12 | 12.63 | 14.90 | 14.69 | 11.86 |
|  |  | **(2.33)** | (3.42) | (3.48) | (3.55) | (3.86) | (3.83) | (3.44) |
| III |  |  | **7.43** | 9.57 | 8.55 | 9.55 | 9.79 | 9.31 |
|  |  |  | **(2.73)** | (3.09) | (2.92) | (3.09) | (3.13) | (3.05) |
| IV |  |  |  | **7.90** | 12.65 | 11.97 | 11.43 | 11.96 |
|  |  |  |  | **(2.81)** | (3.56) | (3.46) | (3.38) | (3.46) |
| V |  |  |  |  | **0.00** | 7.88 | 12.26 | 8.78 |
|  |  |  |  |  | **(0.00)** | (2.81) | (3.50) | (2.96) |
| VI |  |  |  |  |  | **0.00** | 14.84 | 10.19 |
|  |  |  |  |  |  | **(0.00)** | (3.85) | (3.19) |
| VII |  |  |  |  |  |  | **0.00** | 12.60 |
|  |  |  |  |  |  |  | **(0.00)** | **(3.55)** |
| VIII |  |  |  |  |  |  |  | **0.00** |
|  |  |  |  |  |  |  |  | **(0.00)** |

Values in bold figures are intra-cluster distances

Values in parenthesis are √D^2^= D values

**Table -2S-II: Average intra and inter-cluster distances in garden pea during 2020-21**

| Clusters | I | II | III | IV | V |
| --- | --- | --- | --- | --- | --- |
| I | **6.73** | 9.65 | 10.03 | 9.00 | 9.74 |
|  | **(2.59)** | (3.11) | (3.17) | (3.00) | (3.12) |
| II |  | **6.74** | 9.47 | 8.94 | 11.63 |
|  |  | **(2.60)** | (3.08) | (2.99) | (3.41) |
| III |  |  | **8.37** | 10.40 | 13.30 |
|  |  |  | **(2.89)** | (3.22) | (3.65) |
| IV |  |  |  | **0.00** | 12.41 |
|  |  |  |  | **(0.00)** | (3.52) |
| V |  |  |  |  | **0.00** |
|  |  |  |  |  | **(0.00)** |

Values in bold figures are intra-cluster distances

Values in parenthesis are √D^2^= D values

**Table 2S-III: Average intra and inter-cluster distances in garden pea during pooled over years**

| Clusters | I | II | III | IV | V | VI |
| --- | --- | --- | --- | --- | --- | --- |
| I | **8.74** | 10.00 | 12.26 | 13.46 | 11.27 | 12.43 |
|  | **(2.95)** | (3.16) | (3.50) | (3.67) | (3.36) | (3.53) |
| II |  | **0.00** | 7.96 | 12.10 | 11.20 | 11.51 |
|  |  | **(0.00)** | (2.82) | (3.48) | (3.35) | (3.39) |
| III |  |  | **9.17** | 12.88 | 14.32 | 14.93 |
|  |  |  | **(3.02)** | (3.59) | (3.78) | (3.86) |
| IV |  |  |  | **6.95** | 12.44 | 18.09 |
|  |  |  |  | **(2.63)** | (3.53) | (4.25) |
| V |  |  |  |  | **0.00** | 9.76 |
|  |  |  |  |  | **(0.00)** | (3.12) |
| VI |  |  |  |  |  | **0.00** |
|  |  |  |  |  |  | **(0.00)** |
|  |  |  |  |  |  |  |

Values in bold figures are intra-cluster distances

Values in parenthesis are √D^2^= D values

**Table -3S-I: Cluster means for different characters in garden pea during 2019-20**

| **Clusters**  **Traits** | **I** | **II** | **III** | **IV** | **V** | **VI** | **VII** | **VIII** | **Mean** | **Max.** | **Min.** |
| --- | --- | --- | --- | --- | --- | --- | --- | --- | --- | --- | --- |
|  |  |  |  |  |  |  |  |  |  |  |  |
| Days to 50% flowering | 86.64 | 87.00 | 91.90 | 76.53 | 100.67 | 96.33 | 90.00 | 92.00 | 90.13 | 100.67 | 76.53 |
| First flower node | 11.43 | 10.77 | 12.08 | 10.20 | 14.00 | 12.33 | 12.53 | 12.53 | 11.98 | 14.00 | 10.20 |
| Days to first picking | 129.24 | 128.73 | 135.01 | 120.93 | 140.00 | 141.33 | 134.67 | 140.33 | 133.78 | 141.33 | 120.93 |
| Pod length | 10.83 | 11.09 | 10.01 | 8.81 | 10.11 | 8.95 | 10.33 | 12.42 | 10.32 | 12.42 | 8.81 |
| Pod width | 1.80 | 1.75 | 1.74 | 1.93 | 1.95 | 1.69 | 1.92 | 2.11 | 1.86 | 2.11 | 1.69 |
| Seeds per pod | 8.12 | 8.65 | 7.31 | 6.61 | 8.07 | 5.40 | 7.17 | 6.67 | 7.25 | 8.65 | 5.40 |
| Shelling (%) | 47.06 | 47.91 | 46.06 | 43.68 | 44.03 | 46.17 | 46.47 | 43.50 | 45.61 | 47.91 | 43.50 |
| Average pod weight | 5.48 | 5.57 | 4.83 | 4.81 | 5.14 | 4.69 | 4.76 | 6.37 | 5.21 | 6.37 | 4.69 |
| Harvest duration | 26.26 | 25.40 | 21.67 | 27.33 | 19.33 | 18.33 | 26.67 | 18.33 | 22.92 | 27.33 | 18.33 |
| Number of branches per plant | 1.88 | 2.13 | 1.71 | 1.47 | 1.53 | 1.60 | 1.93 | 1.93 | 1.77 | 2.13 | 1.47 |
| Number of nodes per plant | 23.96 | 25.11 | 23.85 | 19.73 | 22.27 | 21.93 | 26.73 | 27.47 | 23.88 | 27.47 | 19.73 |
| Internodal length | 6.67 | 6.72 | 6.65 | 6.37 | 6.01 | 6.27 | 7.11 | 6.89 | 6.59 | 7.11 | 6.01 |
| Plant height | 83.6 | 76.51 | 91.57 | 80.04 | 79.72 | 65.13 | 128 | 84.2 | 86.10 | 128 | 65.13 |
| Number of pods per plant | 15.13 | 19.62 | 13.23 | 11.65 | 14.23 | 8.09 | 14.94 | 10.93 | 13.48 | 19.62 | 8.09 |
| TSS | 16.51 | 16.55 | 15.96 | 16.47 | 17 | 16.73 | 16.27 | 18.77 | 16.78 | 18.77 | 15.96 |
| Ascorbic acid | 25.6 | 26.55 | 25.78 | 27.04 | 22.4 | 26.67 | 25.6 | 25.27 | 25.61 | 27.04 | 22.40 |
| Moisture content | 5.12 | 4.97 | 5.13 | 5.01 | 4.37 | 5.50 | 4.73 | 4.57 | 4.93 | 5.50 | 4.37 |
| Sugars | 5.72 | 6.03 | 6.08 | 5.46 | 5.47 | 5.73 | 4.80 | 6.07 | 5.67 | 6.08 | 4.80 |
| Pod yield per plant | 82.87 | 108.89 | 63.15 | 55.31 | 73.13 | 37.73 | 71.15 | 69.67 | 70.24 | 108.89 | 37.73 |

**Table -3S-II: Cluster means for different characters in garden pea during 2020-21**

| **Clusters**  **Traits** | **I** | **II** | **III** | **IV** | **V** | **Mean** | **Max.** | **Min.** |
| --- | --- | --- | --- | --- | --- | --- | --- | --- |
|  |  |  |  |  |  |  |  |  |
| Days to 50% flowering | 90.49 | 93.31 | 83.28 | 92.67 | 89 | 89.75 | 93.31 | 83.28 |
| First flower node | 11.14 | 11.88 | 9.99 | 13.13 | 11.67 | 11.56 | 13.13 | 9.99 |
| Days to first picking | 124.42 | 128.24 | 120.50 | 123.33 | 128.33 | 124.96 | 128.33 | 120.50 |
| Pod length | 11.37 | 9.80 | 10.19 | 9.20 | 13.37 | 10.79 | 13.37 | 9.20 |
| Pod width | 1.85 | 1.84 | 1.86 | 1.82 | 2.17 | 1.91 | 2.17 | 1.82 |
| Seeds per pod | 8.85 | 7.63 | 7.87 | 7.50 | 7.83 | 7.94 | 8.85 | 7.50 |
| Shelling (%) | 48.83 | 48.09 | 47.06 | 46.27 | 46.07 | 47.26 | 48.83 | 46.07 |
| Average pod weight | 6.06 | 5.12 | 5.38 | 4.72 | 7.39 | 5.73 | 7.39 | 4.72 |
| Harvest duration | 21.31 | 18.30 | 21.61 | 20.67 | 18.00 | 19.98 | 21.61 | 18.00 |
| Number of branches per plant | 1.87 | 1.89 | 1.43 | 1.07 | 2.30 | 1.71 | 2.30 | 1.07 |
| Number of nodes per plant | 24.65 | 25.31 | 20.57 | 17.60 | 28.73 | 23.37 | 28.73 | 17.60 |
| Internodal length | 6.51 | 6.36 | 5.69 | 6.69 | 6.52 | 6.35 | 6.69 | 5.69 |
| Plant height | 70.71 | 73.55 | 65.83 | 83.79 | 72.87 | 73.35 | 83.79 | 65.83 |
| Number of pods per plant | 17.03 | 15.36 | 14.51 | 23.14 | 11.55 | 16.32 | 23.14 | 11.55 |
| TSS | 16.54 | 16.69 | 15.23 | 16.03 | 19.97 | 16.89 | 19.97 | 15.23 |
| Ascorbic acid | 25.89 | 25.71 | 26.08 | 27.73 | 31.67 | 27.42 | 31.67 | 25.71 |
| Moisture content | 4.68 | 4.82 | 5.4 | 6.37 | 4.33 | 5.12 | 6.37 | 4.33 |
| Sugars | 5.86 | 5.8 | 6.05 | 6.73 | 6.33 | 6.15 | 6.73 | 5.80 |
| Pod yield per plant | 102.67 | 77.92 | 76.47 | 108.73 | 85.17 | 90.19 | 108.73 | 76.47 |

**Table -3S-III: Cluster means for different characters in garden pea during pooled years**

| **Clusters**  **Traits** | **I** | **II** | **III** | **IV** | **V** | **VI** | **Mean** | **Max.** | **Min.** |
| --- | --- | --- | --- | --- | --- | --- | --- | --- | --- |
|  |  |  |  |  |  |  |  |  |  |
| Days to 50% flowering | 88.93 | 95.50 | 93.38 | 74.83 | 83.50 | 90.50 | 87.77 | 95.50 | 74.83 |
| First flower node | 11.31 | 12.00 | 12.07 | 9.57 | 10.30 | 12.10 | 11.23 | 12.10 | 9.57 |
| Days to first picking | 127.88 | 133.50 | 131.53 | 115.61 | 123.33 | 134.33 | 127.70 | 134.33 | 115.61 |
| Pod length | 11.17 | 9.82 | 9.68 | 8.47 | 10.40 | 12.90 | 10.41 | 12.90 | 8.47 |
| Pod width | 1.81 | 1.86 | 1.78 | 1.87 | 2.22 | 2.14 | 1.95 | 2.22 | 1.78 |
| Seeds per pod | 8.52 | 7.27 | 7.33 | 6.59 | 7.27 | 7.25 | 7.37 | 8.52 | 6.59 |
| Shelling (%) | 47.73 | 47.60 | 47.22 | 46.01 | 39.17 | 44.78 | 45.42 | 47.73 | 39.17 |
| Average pod weight | 5.75 | 5.60 | 4.82 | 4.59 | 6.58 | 6.88 | 5.70 | 6.88 | 4.59 |
| Harvest duration | 22.92 | 16.67 | 20.37 | 24.33 | 26.17 | 18.17 | 21.44 | 26.17 | 16.67 |
| Number of branches per plant | 1.84 | 1.87 | 1.77 | 1.44 | 1.83 | 2.12 | 1.81 | 2.12 | 1.44 |
| Number of nodes per plant | 24.27 | 26.02 | 24.16 | 19.21 | 22.42 | 28.10 | 24.03 | 28.10 | 19.21 |
| Internodal length | 6.62 | 6.51 | 6.41 | 5.82 | 6.70 | 6.70 | 6.46 | 6.70 | 5.82 |
| Plant height | 78.37 | 74.33 | 82.46 | 69.36 | 73.94 | 78.53 | 76.17 | 82.46 | 69.36 |
| Number of pods per plant | 15.97 | 12.74 | 14.55 | 13.98 | 11.81 | 11.24 | 13.38 | 15.97 | 11.24 |
| TSS | 16.31 | 15.88 | 16.46 | 15.61 | 17.58 | 19.37 | 16.87 | 19.37 | 15.61 |
| Ascorbic acid | 25.75 | 27.03 | 25.93 | 25.01 | 30.23 | 28.47 | 27.07 | 30.23 | 25.01 |
| Moisture content | 4.90 | 5.18 | 4.96 | 5.52 | 4.83 | 4.45 | 4.97 | 5.52 | 4.45 |
| Sugars | 5.87 | 5.43 | 6.00 | 5.31 | 5.97 | 6.20 | 5.80 | 6.20 | 5.31 |
| Pod yield per plant | 91.82 | 71.04 | 69.81 | 63.82 | 77.59 | 77.42 | 75.25 | 91.32 | 63.82 |
|  |  |  |  |  |  |  |  |  |  |
