# Supplementary figures and images for: Morpho-molecular genetic diversity and population structure analysis in garden pea (Pisum sativum L.) genotypes using simple sequence repeat markers
Source: PLoS One. 2022 Sep 16;17(9):e0273499. doi: 10.1371/journal.pone.0273499 (PMC9480992; doi:10.1371/journal.pone.0273499)

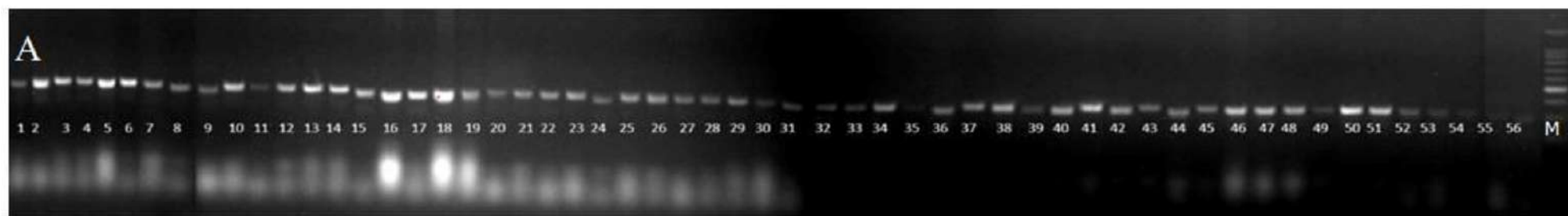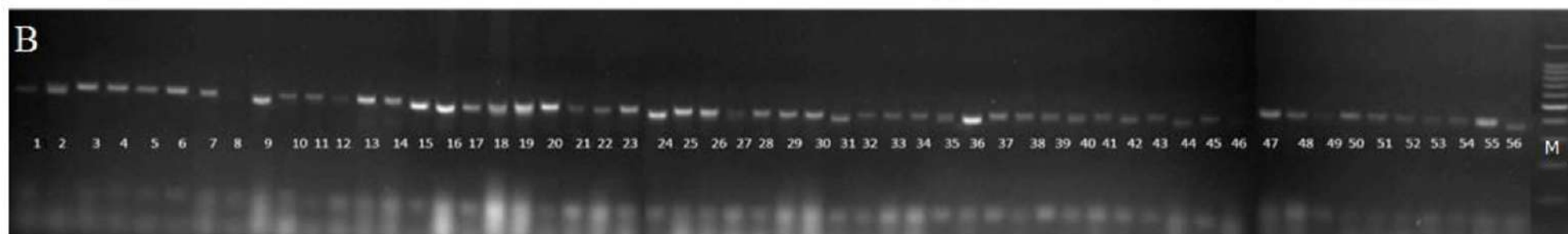

Supplement: S1 Raw image — (PDF) [file pone.0273499.s010.pdf]
