# Supplementary material for: Morpho-molecular genetic diversity and population structure analysis in garden pea (Pisum sativum L.) genotypes using simple sequence repeat markers
Source: PLoS One. 2022 Sep 16;17(9):e0273499. doi: 10.1371/journal.pone.0273499 (PMC9480992; doi:10.1371/journal.pone.0273499)
Supplement: Attachment — Submitted filename: Response to Reviewers.doc [file pone.0273499.s012.doc]

**Sub and Reference:** Point wise responses to the comments by the academic editor/ reviewers

I have revised our manuscript (PONE-D-22-00070) as per comments and suggestions raised by Academic Editor and Reviewer#1, Reviewer#2 and Reviewer#3. I used the Track Changes for revision to highlight the additions/deletions/changes. Point wise responses to the comments/ suggestions are as below:

**Academic Editor:**

**General 1:** After careful consideration, we feel that it has merit but does not fully meet PLOS ONE’s publication criteria as it currently stands. Therefore, we invite you to submit a revised version of the manuscript that addresses the points raised during the review process.

**Response:** Dear Sir, I have revised the manuscript as per the comments and suggestions. I am greatly thankful to you for positive decision on this manuscript.

**Reviewer#1**

**Comment 1:** The innovation in the study with previous studies.

**Response:** In the current study, we compared the combination of agro-morphological approach together with a molecular evaluation in order to gain a better knowledge on available garden pea genotypes. As results which we got from the project, both stages of characterization are crucial for a better understanding of the genetic variability.

In the present study, we also screened the genotypes against powdery mildew disease, and reported diverse genotypes (susceptible and resistant based on cluster analysis) for development of mapping population.

Addition at: Line 32-36, 75-79, 85-87, 353-360

**Comments 2:** The future prospect and output of the study in which the study is based

**Response:** Presented in discussion and conclusion section.

Line 379-383, 461-467

**Comments 3:** What is the take home message of the study. That means any genotype most promising or least promising for future studies and why?

**Response:** Presented at Line 345-365, 379-383, 398-404, 451-454, 461-467

**Reviewer#2**

**General: T**he manuscript entitled “Morpho-molecular genetic diversity and population structure analysis in garden pea (Pisum sativum L.) genotypes using simple sequence repeat markers" by Sharma et al. is well written and organized. I feel that the results provided by the authors will contribute to progress in the future garden pea breeding programs. As such, the manuscript contains a large amount of data. In general, the paper presents results that are novel and merits publication. So, I strongly recommend the manuscript and can be accepted for publication

**Response:** I am greatly thankful to the reviewer to recommending our research for publication in reputed journal “*PLoS ONE*”.

**Comment 1:** Figures 3 and 6 are not clearly visible.

**Response:** Added new figure with clear visibility.

**Reviewer#3**

**General comment 1:** My major concern is the rationale and relevance of the such study.

**Response:** In the current study, we compared the combination of agro-morphological approach together with a molecular evaluation in order to gain a better knowledge on available garden pea genotypes. Meanwhile, information on characterization of garden pea genotypes using combination of agro-morphological approach together with a molecular evaluation is scare.

**General comment 2:** Overall, this study looks highly repetitive with the previous ones (that authors have mentioned frequently in the discussion section for e.g. page 17, line 313-315; line 318; page 18-19 line 358-362 and many more places)

**Response:** The cited studied to support our results were on diversity analysis using agronomic traits or some of them use molecular approach. Some of them are on other crops like Cauliflower or Maize to better support of statements. There are limited studies on both approaches i.e. agro-morphological along with molecular evaluation.

**General comment 3:** The sentences are often vague and at places incomplete (line 294).

**Response:** Corrected all incomplete sentences throughout the manuscript.

**General comment 4:** The presentation of results is complex while the outcome of the study is very generalised.

**Response:** Added outcome of the study in discussion section as well under conclusion part.

**General comment 5:** Also, why morphological markers were chosen in study when it was clear at the first place that they are highly variable and do not help in genetic diversity analysis?

**Response:** We stated that both the strategies are useful for diversity analysis. Because when, we compared the groups formed by agro-morphological and molecular data, no genotypes were observed, indicating that both stages of characterization are crucial for a better understanding of the genetic variability.
