# Supplementary material for: Morpho-molecular genetic diversity and population structure analysis in garden pea (Pisum sativum L.) genotypes using simple sequence repeat markers
Source: PLoS One. 2022 Sep 16;17(9):e0273499. doi: 10.1371/journal.pone.0273499 (PMC9480992; doi:10.1371/journal.pone.0273499)
Supplement: Attachment — Submitted filename: Reviewer comment.docx [file pone.0273499.s011.docx]

The manuscript entitled “Morpho-molecular genetic diversity and population structure analysis in garden pea (*Pisum sativum* L.) genotypes using simple sequence repeat markers" by Sharma et al. is well written and organized.

Herein, the authors have demonstrated the genetic diversity among 56 garden pea genotypes using morphological descriptors, quantitative traits, and simple sequence repeat markers. I feel that the results provided by the authors will contribute to progress in the future garden pea breeding programs. As such, the manuscript contains a large amount of data. In general, the paper presents results that are novel and merits publication.

So, I strongly recommend the manuscript and can be accepted for publication after incorporating corrections as suggested below:

1. Figures 3 and 6 are not clearly visible.
